# Perforated Carcinoma in the Gastric Remnant: A Case of Conservative Treatment Prior to Successful Curative R0 Resection

**DOI:** 10.1155/2016/4091952

**Published:** 2016-08-29

**Authors:** Ken Yuu, Hiroshi Kawashima, Sho Toyoda, Satoshi Okumura, Kansuke Yamamoto, Naoto Mizumura, Aya Ito, Hiromitsu Maehira, Atsuo Imagawa, Masao Ogawa, Masayasu Kawasaki, Masao Kameyama

**Affiliations:** Department of Surgery, Bell Land General Hospital, 500-3 Higashiyama, Naka-ku, Sakai, Osaka 599-8247, Japan

## Abstract

An 80-year-old man who had undergone distal gastrectomy and Billroth-II gastrojejunostomy 38 years previously, for a benign gastric ulcer, was diagnosed with remnant gastric cancer based on upper gastrointestinal endoscopy findings. He presented at our emergency department with acute-onset epigastric pain due to perforated remnant gastric cancer. Conservative medical management was selected, including nasogastric tube insertion, antibiotics, and proton pump inhibitors, because his peritonitis was limited to his epigastrium and his general condition was stable. Twenty-one days after the perforation occurred, curative total remnant gastrectomy and D2 lymphadenectomy were performed. Adhesion between the lateral segment of the liver and the dissected lesser curvature of the gastric remnant may have contributed to the peritonitis in this case, which was limited to the epigastrium. This is the first report of perforated remnant gastric cancer in which conservative treatment was effective prior to curative resection. The protocol reported here may be of use to other clinicians who may encounter this clinical entity in their practices.

## 1. Introduction

Gastric perforation is one of the most frequent causes of acute abdominal pain [1]. The main cause of gastric perforation is gastric ulcer, but approximately 10% of cases are caused by gastric cancer [2]. In the past, emergent one-stage gastrectomy was performed for most cases of gastric perforation with diffuse peritonitis, regardless of whether the disease was benign or malignant [3]. However, one-stage gastrectomy has been found to be associated with high mortality rates (0–50%) [3]. Moreover, sufficient lymph node dissection is difficult to achieve during emergency surgery for perforated gastric cancer, and this may impair long-term survival due to the risk of recurrence [3]. In patients in a poor clinical condition, simple closure and omental patch repair are suitable. If the perforation is caused by cancer, however, the risk of secondary leakage due to reperforation cannot be ignored [4]. Initial conservative treatment has been performed in patients with limited peritonitis, and subsequent elective gastrectomy can be planned following recovery from peritonitis. The standard treatment for perforated gastric cancer has not been established.

Remnant gastric cancer was first described in 1922 by Balfour [5]. The incidence of metachronous remnant gastric cancer has been reported as 1.0–3.0%. Although mass screening has improved the early detection rates of gastric cancer in Korea and Japan, remnant gastric cancer is still frequently found at the more advanced stages at the time of detection. Here, we present a case of perforated remnant gastric cancer that was initially treated with conservative treatment. After the patient recovered from peritonitis, total remnant gastrectomy with D2 lymph node dissection was performed and curative R0 resection was achieved.

## 2. Case Presentation

**Figure 1:**
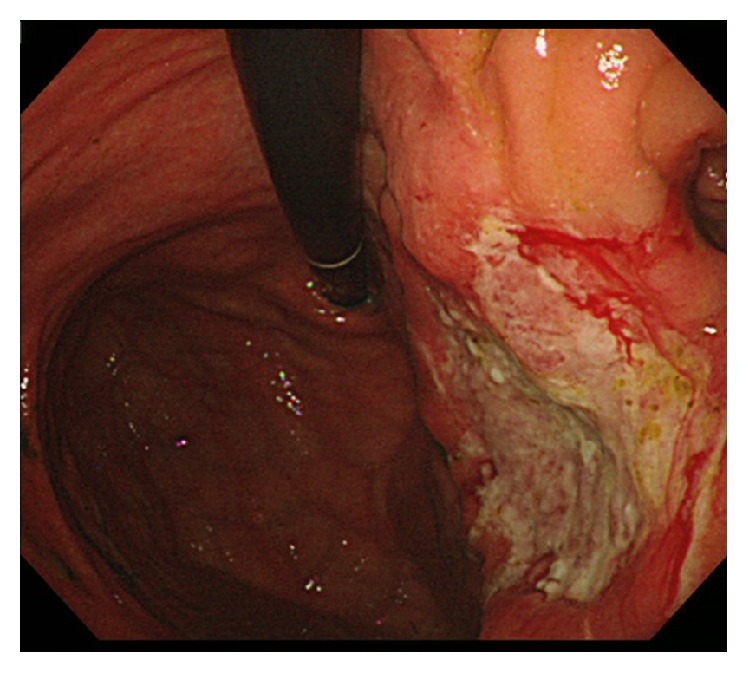
Upper gastrointestinal fiberscopy findings. There was the ulcerated tumor about 4 cm in size (type 3). The tumor was found at the remnant stomach and invaded to the anastomotic site of Billroth-II gastrojejunostomy.

An 80-year-old man was diagnosed with advanced remnant gastric cancer detected using upper gastrointestinal fiberscopy. He had undergone gastrectomy for a benign gastric ulcer 38 years previously, and Billroth-II gastrojejunostomy antecolic reconstruction was performed after gastrectomy. There was an upper-middle operative scar, about 20 cm in length, on his abdomen. The concentrations of the tumor markers CEA, CA 19-9, and CA 125 were 6.0 ng/mL (<5.0 ng/mL), 408 U/mL (<37.0 U/mL), and 66.3 U/mL (<35.0 U/mL), respectively. Upper gastrointestinal fiberscopy for annual follow-up revealed a type 3 shaped tumor, 4.0 cm in size, located in the gastric remnant near the gastrojejunostomy (Figure 1). Examination of a biopsy specimen showed well-differentiated adenocarcinoma. A clinical diagnosis of advanced gastric cancer (B-38-O, T4a [SE] N0 M0, Stage IIB) was made according to the Japanese Classification of Gastric Carcinoma following distal gastrectomy [6].

**Figure 2:**
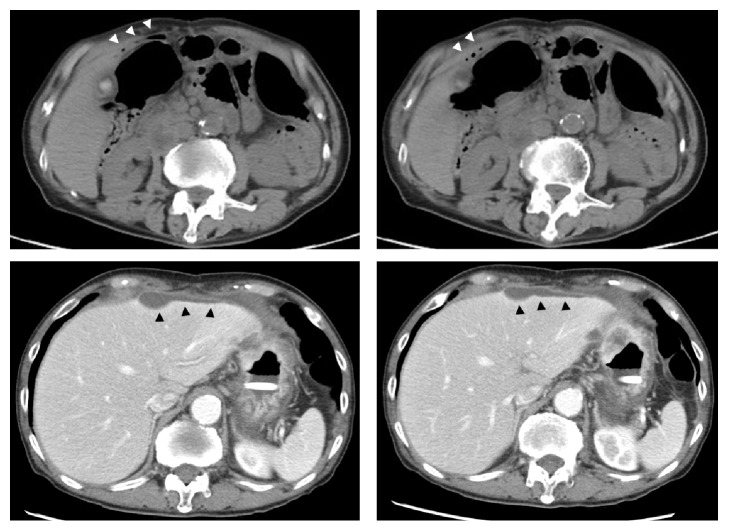
Computed tomography of abdomen and pelvis, showing abnormal pneumoperitoneum (white arrowhead) and limited ascites (black arrowhead).

When the patient was waiting to undergo elective gastrectomy with D2 lymph node dissection, he presented at our emergency department with acute-onset epigastric pain. Computed tomography (CT) confirmed the presence of free air and limited ascites (Figure 2). The leucocyte count (160 × 10^2^/*μ*L) and levels of C-reactive protein (12.0 mg/dL), blood urea nitrogen, and creatinine were slightly elevated. He was fully conscious with mental clarity, and no shock had developed. His blood pressure and heart rate were normal.

**Figure 3:**
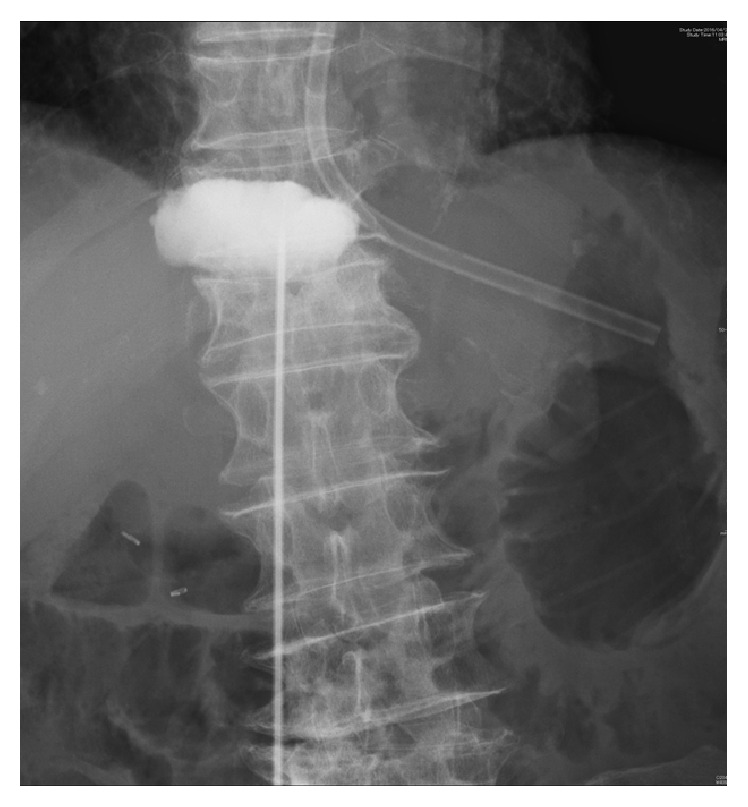
Percutaneous drainage was performed 3 days after perforation. Pale yellow ascitic fluid was drained. The result of peritoneal lavage cytology was negative.

Considering the general condition of the patient due to his limited peritonitis and the complexity involved with curative gastrectomy with* en bloc* D2 lymph node dissection, conservative treatment was selected. The conservative treatment included nasogastric tube drainage, proton pump inhibitors, antibiotics, and percutaneous drainage (Figure 3). Approximately 60 mL of pale yellow ascitic fluid was drained and then examined pathologically. The result of peritoneal lavage cytology was negative. The abdominal symptoms improved after 3 days, and the patient was able to tolerate oral feeding 7 days after the perforation was diagnosed.

**Figure 4:**
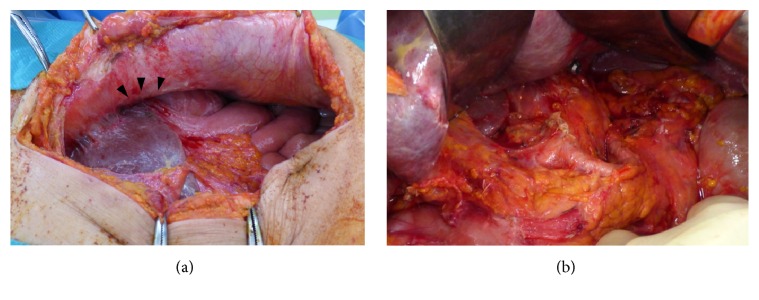
(a) The tightest adhesion (black arrowhead) between the lateral segment of the liver and the lesser curvature of the gastric remnant due to previous surgery and the perforation. (b) Curative gastrectomy with D2 lymphadenectomy was performed.

After recovering from peritonitis due to perforation of the carcinoma in the gastric remnant, radical total remnant gastrectomy with D2 lymph node dissection and Roux-en-Y esophagojejunostomy were performed 21 days after the perforation (Figure 4(b)). No peritoneal metastasis was noted during surgery. The results of peritoneal lavage cytology were negative at this point. The patient experienced an uneventful postoperative recovery and was discharged in good health 12 days after surgery.

**Figure 5:**
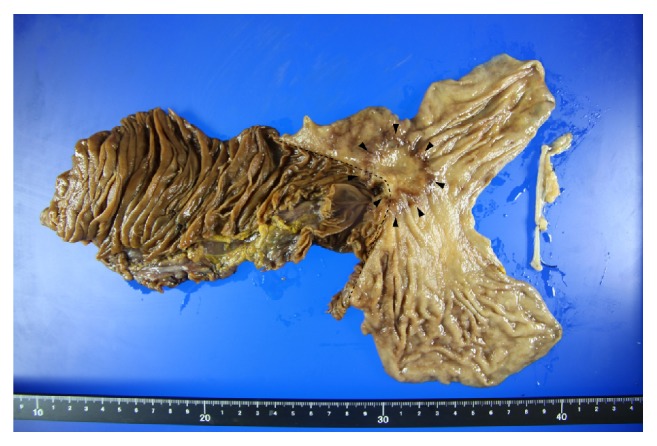
Resected specimen. The resected stomach contained an infiltrative-ulcerative type tumor, 25 × 25 mm in size (black arrowhead). The black dote line showed the gastrojejunostomy at initial gastrectomy.

The resected stomach contained an infiltrative-ulcerative type tumor that was 25 × 25 mm in size (Figure 5). Histological examination revealed well-differentiated adenocarcinoma extending to a depth beyond the serosa, with lymph node metastasis (number 3a), that was pathologically classified as Stage IIIB.

## 3. Discussion

Above, we have described an 80-year-old Japanese man who underwent combined modality therapy on the perforated remnant gastric cancer. This is the first reported case of conservative treatment and radical gastrectomy for perforated remnant gastric cancer. In this case, remnant total gastrectomy with D2 lymphadenectomy was completed and R0 resection was achieved.

Remnant gastric cancer was originally defined as cancer detected in the gastric remnant after distal gastrectomy in benign cases. The treatment for remnant gastric cancer includes surgical treatment, radiation therapy, and chemotherapy, which are all similar to the methods used for primary gastric cancers. Surgery is known to be the only curative method, and complete resection of the remnant stomach and D2 lymphadenectomy are commonly performed [7]. Although mass screening has improved the early detection rate of gastric cancers in Japan and Korea, remnant gastric cancer is still often at an advanced stage when it is detected. Furthermore, anatomical alterations, intraabdominal adhesions, and the frequent combined resection of other organs render surgery for remnant gastric cancer difficult. Hu et al. [7] reported that the 5-year survival rate of patients undergoing curative resection was higher than that of those who did not undergo this treatment. Lee et al. [8] reported that radical resection is very important for improving the survival rate of patients with remnant gastric cancer. In the present case, we detected tight adhesions between the lateral segment of the liver and the lesser curvature of the gastric remnant. The lateral segment of the liver was preserved, because the adhesion was deemed to be the result of previous surgery and the inflammatory adhesion due to perforation.

Gastrointestinal perforation is often suspected based on the presence of certain clinical symptoms (e.g., abdominal rebound tenderness or muscular guarding with high-grade fever) and confirmed by imaging modalities including abdominal CT. When diffuse peritonitis due to gastrointestinal perforation is diagnosed, emergent surgery is necessary without further detailed preoperative examinations. It has been reported that about 10–16% of all gastric perforations are caused by gastric cancer [9, 10]. Several reports of perforated gastric cancer have demonstrated significantly better prognoses for patients who undergo curative resection than those who undergo noncurative resection [10–12]. Furthermore, multivariate analysis has supported the importance of R0 resection with radical surgery for good prognosis. Several reports describe patients who did not undergo R0 resection with radical surgery associated with lymph node dissection as the initial surgery due to diffuse peritonitis or insufficient examination [13]. Lehnert et al. [10] recommend that the initial surgery should be directed toward the treatment of peritonitis and that radical oncological surgery for gastric cancer should be planned following the patient's recovery. Hata et al. [14] reported that the rates of R0 resection and D2 lymph node dissection were significantly higher in two-stage gastrectomy, in which the initial treatment of peritonitis is followed by elective gastrectomy, than in patients who underwent emergent one-stage gastrectomy. In addition, the mortality rate of patients treated with two-stage gastrectomy cases was significantly lower than that in those treated with one-stage gastrectomy. In the present case, conservative treatment was used with the aim of eventually performing successful surgery. There are no reports investigating the interval between initial treatment for peritonitis and curative surgery; however, based on our knowledge, we believe that effective conservative treatment may allow earlier curative surgery.

Hu et al. [7] reported that, during surgery for remnant gastric cancer, the rate of the combined resection of adjacent organs can be increased by adhesions to the adjacent organs. The tightest adhesion seen in the present case was between the lateral segment of the liver and the gastric remnant (Figure 4(a)). As a reason for this, we suspect incomplete cure of diffuse peritonitis by conservative treatment. The patients with a history of gastrectomy might not develop diffuse peritonitis due to remnant gastric perforation. Percutaneous drainage is effective for treating mild ascites and mild inflammation resulting from perforation. Percutaneous drainage has local effects but is less invasive than the other options. It is important to observe the course of treatment carefully. If percutaneous drainage is insufficient, open drainage should be considered [15]. This strategy is recommended for remnant gastric cancer, and the avoidance of invasive procedures allows radical and curative gastrectomy to be performed immediately.

It has been reported that, for patients with remnant gastric cancer, the disease tends to be at a high stage at detection, and lymph node metastasis is common in these patients [16, 17]. Therefore, it is very important to understand the characteristics of remnant gastric cancer and to determine its prognostic factors in order to determine the optimal treatment method. This is the first report of perforated remnant gastric cancer treated by conservative treatment followed by curative gastrectomy. This treatment combination is effective for perforated remnant gastric cancer and should be assessed by other clinicians in future studies.
